# Single locus genotyping to track *Leishmania donovani* in the Indian subcontinent: Application in Nepal

**DOI:** 10.1371/journal.pntd.0005420

**Published:** 2017-03-01

**Authors:** Keshav Rai, Narayan Raj Bhattarai, Manu Vanaerschot, Hideo Imamura, Gebreyohans Gebru, Basudha Khanal, Suman Rijal, Marleen Boelaert, Chiranjib Pal, Prahlad Karki, Jean-Claude Dujardin, Gert Van der Auwera

**Affiliations:** 1 Department of Microbiology, B.P. Koirala Institute of Health Sciences, Dharan, Nepal; 2 Department of Zoology, West Bengal State University, Barasat, West Bengal, India; 3 Department of Microbiology and Immunology, Columbia University College of Physicians and Surgeons, New York, NY, United States of America; 4 Department of Biomedical Sciences, Institute of Tropical Medicine, Antwerp, Belgium; 5 Department of Animal Science, College of Agriculture, Aksum University, Aksum, Ethiopia; 6 Department of Internal Medicine, B.P. Koirala Institute of Health Sciences, Dharan, Nepal; 7 Department of Public Health, Institute of Tropical Medicine, Antwerp, Belgium; 8 Department of Biomedical Sciences, University of Antwerp, Belgium; US Food and Drug Administration, UNITED STATES

## Abstract

**Background:**

We designed a straightforward method for discriminating circulating *Leishmania* populations in the Indian subcontinent (ISC). Research on transmission dynamics of visceral leishmaniasis (VL, or Kala-azar) was recently identified as one of the key research priorities for elimination of the disease in the ISC. VL in Bangladesh, India, and Nepal is caused by genetically homogeneous populations of *Leishmania donovani* parasites, transmitted by female sandflies. Classical methods to study diversity of these protozoa in other regions of the world, such as microsatellite typing, have proven of little use in the area, as they are not able to discriminate most genotypes. Recently, whole genome sequencing (WGS) so far identified 10 different populations termed ISC001-ISC010.

**Methodology / Principle findings:**

As an alternative to WGS for epidemiological or clinical studies, we designed assays based on PCR amplification followed by dideoxynucleotide sequencing for identification of the non-recombinant genotypes ISC001 up to ISC007. These assays were applied on 106 parasite isolates collected in Nepal between 2011 and 2014. Combined with data from WGS on strains collected in the period 2002–2011, we provide a proof-of-principle for the application of genotyping to study treatment outcome, and differential geographic distribution.

**Conclusions / Significance:**

Our method can aid in epidemiological follow-up of visceral leishmaniasis in the Indian subcontinent, a necessity in the frame of the Kala-azar elimination initiative in the region.

## Author summary

Visceral Leishmaniasis (VL) or Kala-azar is a life-threatening neglected tropical disease that annually affects half a million people worldwide. In the Indian subcontinent (India, Nepal, Bangladesh), the disease is caused by infection with the protozoan parasite *Leishmania donovani*, which is transmitted by female sand flies. Currently, the Kala-azar elimination program aims at reducing the number of VL cases in the region to less than 1 in 10.000 at upazila, sub-district and district level in Bangladesh, India, and Nepal respectively. In support of this program, tools for tracking *L*. *donovani* populations are essential, because these allow monitoring geographic spread over time. However, the parasite populations in the region are highly homogeneous, requiring sequencing of the entire genome to gather sufficient information for discriminating them. Because whole genome sequencing (WGS) is impractical for large-scale use, we designed a simple alternative to identify the WGS-genotypes. Our method is based on PCR amplification followed by sequencing of one particular locus, diagnostic of each population. We provide proof-of-principle that our method can be used to track parasite populations over time, and to correlate them with clinical parameters. We believe that our assay can support the Kala-azar control efforts in the Indian subcontinent.

## Introduction

Visceral Leishmaniasis (VL) is a neglected tropical disease caused by parasites of the *Leishmania donovani* species complex, which are transmitted by the bite of phlebotomine sand flies. WHO estimates that annually, 300 million people worldwide are at risk of VL [1]. In the Indian subcontinent (ISC), including Bangladesh, India, and Nepal, each year an estimated 237,500 new cases occurred, of which 4,450 in Nepal [2]. Together this accounts for 80% of all new VL cases worldwide. However, recently VL incidence is declining in the Indian subcontinent, and the numbers of new reported cases have dropped to 735, 9241, and 335 respectively in the aforementioned countries in 2014 [3]. Monitoring/tracking the genetic diversity of the parasites in these endemic regions over time is essential to understand transmission dynamics. This can help to evaluate the effect of both preventive and curative intervention programs, to monitor progress towards elimination, and to study the dynamics of variants associated with specific phenotypes.

Several molecular assays for identifying populations of *L*. *donovani* have been developed. Among these are sequencing of ribosomal loci [4], multi-locus microsatellite typing [5], multilocus sequence typing [6], amplified fragment length polymorphism analysis [7], and kinetoplast minicircle DNA RFLP (restriction fragment length polymorphism) analysis [8,9]. These methods are capable of discriminating a number of genotypes, but are less effective in recently evolving epidemic parasite populations which are relatively homogeneous, such as *L*. *donovani* in the ISC. A phylogenomic study, based on whole genome sequences of over 200 clinical ISC parasite isolates, allowed documenting genetic diversity on a much finer scale [10]. Based on single point mutations (SNPs), ten major ISC populations (named ISC001-ISC010) could be defined. Seven of them (ISC001-ISC007) represented congruent monophyletic groups, while the remaining three (ISC008-ISC010) contained mixed signatures of these.

Because whole genome sequencing (WGS) cannot currently be applied in all laboratories, our goal was to develop simpler molecular assays for discriminating ISC001-ISC007 in individual isolates, based on PCR amplification followed by amplicon sequencing, further called ISC single locus genotyping (ISC-SLG). This will allow application in epidemiological surveys and transmission studies. In case of genotype ISC005, such assay could also have a clinical relevance, as this genotype was shown to correlate with antimonial drug resistance and treatment failure [10]. After development of the assay and evaluation of its analytical performance, we applied it to Nepalese isolates that were collected in the years following the WGS survey, and analyzed the spread of genotypes over time and space.

## Methods

### Ethics statement

Ethical clearance was obtained from the institutional review boards of the Nepal Health Research Council, Kathmandu, Nepal and the corresponding body of the Institute of Tropical Medicine, Antwerp, Belgium.

### Sample collection and parasite isolation

The sample collection consisted of a total of 204 *Leishmania* isolates from 195 confirmed VL patients who presented between 2002 and July 2014 at the B. P. Koirala Institute of Health Sciences (BPKIHS), a tertiary care medical center in Dharan, Nepal. The clinical criteria were fever for more than 2 weeks, combined with hepatomegaly and/or splenomegaly. The laboratory criteria were a positive rK39 rapid diagnostic test (InBiOS, Cat. nr. INS015) [11,12], and bone marrow smear positivity. Written informed consent was obtained from patients, or from parents or guardians in case of children.

Detailed geographical and clinical information of each patient is provided in S1 Database. Among the 195 patients, 9 were treated twice because they either did not respond to the first treatment, or relapsed. The period during which the second treatment was administered is referred to as second episode, hence our study included 204 disease episodes. Of these, treatment history of 26 was not traceable. The remaining episodes were treated with antimonials (Sodium stibogluconate, SSG), Miltefosine (MIL), or Amphotericin B (AmB) in 33, 85, and 57 cases, respectively, while in 3 cases treatment was not completed. Treatment failure was recorded for 42 episodes, meaning that patients either did not respond to treatment (persisting clinical signs and symptoms of VL, positive bone marrow smear after treatment), or they relapsed after initial cure (reappearance of disease symptoms and/or positive bone marrow smear during 12 months follow-up). Of these 42 episodes, SSG was used in 10, MIL in 29, and AmB in 3 cases.

Parasite promastigotes were derived from bone marrow aspirates of VL patients by culturing in Tobie’s blood agar medium with Locke’s overlay [13], with 200 IU/ml penicillin and 200 μg/ml streptomycin. Once the parasites were fully grown from the clinical material, they were transferred to M199 (Sigma-Aldrich, cat. nr. 2520) with 20% fetal calf serum (Invitrogen, cat. nr. 10270). The parasite cultures were grown to late logarithmic growth phase and cryopreserved at -80˚C with 10% sterile glycerol.

### Parasite DNA extraction and species identification

A total of 204 parasite cultures were isolated at BPKIHS, one for each disease episode. Out of these, 98 had been previously analyzed by WGS [10], while 106 were genotyped in this study with ISC-SLG. DNA was extracted from parasite cultures using the QiaAmp DNA mini kit (Qiagen, www.qiagen.com). Parasites in late logarithmic growth phase were washed thrice with sterile PBS solution and DNA was eluted in 200 μL AE buffer. DNA concentration and purity was verified by spectrophotometric measurement with the NanoDrop 2000 (Thermo Scientific, Waltham, MA, USA). The species of *L*. *donovani* was confirmed using PCR-RFLP analysis of the heat-shock protein 70 gene (*hsp70*). The fragments referred to as HSP70-N [14] were digested with restriction enzymes *Hin*cII (the isoschizomer of *Hin*dII) [15], and *Mlu*I.

### Development of ISC-SLG

Previous WGS analysis of *L*. *donovani* from ISC identified 10 populations [10], seven of which (ISC001-ISC007) being characterized by a unique combination of apomorphic homozygous SNPs or INDELs (insertions/deletions). The remaining three (ISC008-ISC010) represented composite genotypes. For each of the genotypes ISC001-ISC007, a unique apomorphic homozygous SNP or INDEL was selected for designing a specific assay. PCR primers were chosen to amplify about 500 nucleotides flanking each SNP/insertion at both sides. The same primers were used for the amplicon sequencing.

### PCR amplification for ISC-SLG

PCR assays were done in 50 μl final reaction volume which contained 1x PCR buffer with a total of 2 mM MgCl_2_, 200 μM of each dNTP, 1 μM of each primer, and 1.5 units of HotStarTaq Plus DNA polymerase (Qiagen, Cat. nr. 203605). Finally, 0.1 to 1 ng of *L*. *donovani* DNA was added. Since SNPs of ISC001 and ISC002 were located in a high GC% locus, 1x Q-solution was used in both these PCRs to decrease secondary structures. The thermo-cycling program was (i) initial denaturation at 95°C for 5 minutes; (ii) 36 cycles of denaturation at 94 °C for 1 minute, annealing at 60°C for 30 seconds and extension at 72°C for 45 seconds; and (iii) final extension at 72°C for 10 minutes. In addition, two positive controls (1 ng and 0.1 pg parasite DNA of MHOM/NP/2003/BPK282/0cl4) and two no-template controls were included in each experiment. The amplified PCR-products were verified on a 2% agarose gel prior to sequencing.

The analytical sensitivity was determined for each PCR using reference strain MHOM/NP/2003/BPK282/0cl4. Ten-fold DNA dilution series in water were examined, ranging from 20 ng to 0.2 pg added as template. The concentration of the reference DNA was determined using spectrophotometric measurements with the Nanodrop machine (www.nanodrop.com).

### DNA sequence analysis

PCR amplicons were shipped to MACROGEN (www.macrogen.com, Seoul, South Korea) for capillary sequencing (ABI3730XL DNA Analyzer, Applied Biosystems) with either the forward or reverse PCR primer. Chromatogram trace files were aligned with the corresponding reference sequences from WGS data in order to identify the SNPs or insertion in the amplicon. Because we sequenced only one strand, we ensured having an excellent quality read at the nucleotide position in question, and no insertions or deletions outside the query area when aligning to the reference sequence.

### Analysis

GPS coordinates of the villages where patients were living were taken from Google maps (www.google.com.np/maps) [16] and elevations were extracted from the R3.0.3 software (www.R-project.org) [17] using the “rgbif” package. Bubble plots were made with R3.0.3 using the “ggplot2” package. GPS plots were generated with QGIS version 2.8.7-Wien (www.qgis.org) [18] and Google earth (www.google.com/earth) [19].

## Results

### Assay development

For each of the WGS-defined populations ISC001-ISC004 and ISC006-ISC007, a unique homozygous SNP was selected: each SNP was thus an apomorphic character for a given population, also not found in ISC005 or in any of the composite genotypes ISC008-ISC010. In the case of ISC005, an apomorphic two-nucleotide insertion (GA) in the Aquaglyceroporin-1 gene was selected. Table 1 lists all selected SNPs and INDELs, with their characteristics and chromosome location.

**Table 1:** Genetic markers of ISC-SLG.

Genotype	Mutation Type*	Chromosome	Position^@^	Gene ID^@^	Amino Acid change^#^	REFERENCE base°	SNP/INDEL^$^
ISC001	Non-syn	Ld31	1139549	LdBPK_312340.1	D259G	T	C
ISC002	Syn	Ld31	34009	LdBPK_310140.1	S324	C	A
ISC003	Non-syn	Ld36	526311	LdBPK_361470.1	H56Y	G	A
ISC004	Non-syn	Ld35	1001055	LdBPK_352450.1	V1343M	G	A
ISC005	INDEL	Ld31	7735	LdBPK_310030.1	Stop	-	GA
ISC006	NCD	Ld33	364481	NA	NA	A	G
ISC007	Non-syn	Ld16	130313	LdBPK_160390.1	R77K	C	T

* Non-syn = Non-synonymous SNP, Syn = Synonymous SNP, NCD = Non-coding, INDEL = Insertion/Deletion.

@ Annotation in database of GeneDB Ldonovani_BPK282A1_v1 (http://www.genedb.org/web-artemis, last accessed Aug 15, 2016), NA = Not applicable.

# Stop = Stop codon, NA = Not applicable.

° REFERENCE = isolate MHOM/NP/2003/BPK282/0cl4.

^**$**^ SNP = single base change relative to reference, INDEL = Insertion relative to reference.

**Fig 1:**
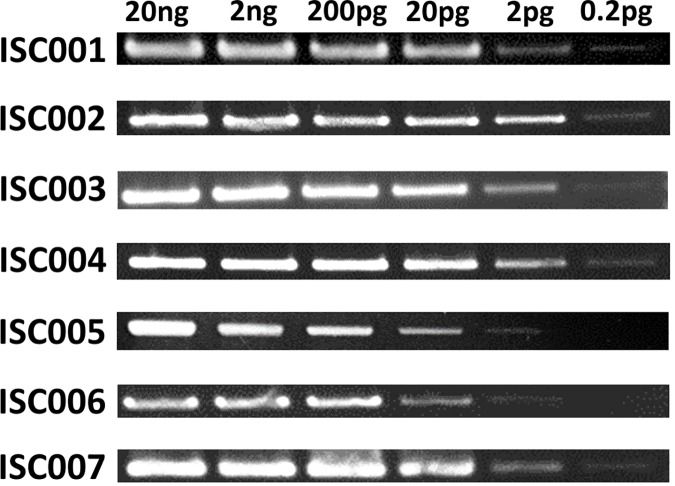
Analytical sensitivity of seven PCRs (Table 2), analyzed on agarose gel. Only the relevant part of the gels is shown. *L*. *donovani* strain MHOM/NP/2003/BPK282/0cl4 DNA was used. The DNA amount used per reaction ranging from 20 ng to 0.2 pg, is indicated on top. The ISC-SLG PCRs are indicated on the left.

The PCR primers that were designed to amplify the specific SNP/INDEL positions with their flanking regions are given in Table 2. Also indicated are the PCR primer that was used for sequencing, and the analytical sensitivity as determined on dilution series of parasite DNA. The analytical sensitivity is illustrated in Fig 1 and ranges between 2 pg and 0.2 pg.

**Table 2:** PCR primers for ISC-SLG.

Genotype	Primer	Sequence	Amplicon size (bp)	Sensitivity
**ISC001**	Ld31_p1139_F2*	CGGTGAGGAATCGCGGCATAG	451	0.2 pg
	Ld31_p1139_R2	GCAGCGCCTTCTTGACGTGATG		
**ISC002**	Ld31_p3400_F1*	TTCAGCTCCGCAAAGGTCGC	357	0.2 pg
	Ld31_p3400_R1	CCGCATGAGCATGCCCAGTC		
**ISC003**	Ld36_p5263_F1*	TGCGGGGTGAAAGGCATCCTG	402	0.2 pg
	Ld36_p5263_R1	CATGCCGGACACTGACCCGT		
**ISC004**	Ld35_p1001_F1*	TCCAACCCTCGTGGCGCAAG	404	0.2 pg
	Ld35_p1001_R1	ACACCCACACCGTGCGATCC		
**ISC005**	AQP_IND_F1*	CGGCTTGTAGTTGACGGCGGGAG	655	2 pg
	AQP_IND_R1	CATGGACAAGGAGGACCCGGAAGG		
**ISC006**	Ld33_p3644_F1	CCCGTGCGTGAAGGCGAACATTT	630	2 pg
	Ld33_p3644_R1*	GTCAGTGTGCAGGGGCGACGG		
**ISC007**	Ld16_p1303_F2	CTCGCTGCCCACCAGACTTTCT	245	0.2 pg
	Ld16_p1303_R2*	CGGACAATTTCGCTACTGGTGCT		

*PCR primer that was used for sequencing.

### Genotyping of clinical isolates

A total of 204 clinical parasite isolates of *L*. *donovani* obtained in Nepal were included in this study (detailed information in S1 Database), 98 of which were collected between 2002 and 2011 and were previously analyzed with WGS [10]. For the present study, we used ISC-SLG to classify the remaining 106 isolates collected between mid-2011 and 2014 in genotypes ISC001 (n = 21), ISC003 (n = 9), ISC004 (n = 8), ISC005 (n = 8), ISC006 (n = 16), and ISC007 (n = 0), while 44 could not be classified. This was done in a sequential manner, whereby the assays for the most common Nepalese genotypes were performed first. Isolates that could not be assigned to a genotype at this stage were further analyzed for less common genotypes, and so on, till all six assays were used. If an isolate could not be categorized after running the six assays, it was listed as “unclassified genotype”. Even though we also developed ISC-SLG for ISC002, we did not test for this genotype as it was so far only reported from Bangladesh [10], and migration between both countries is restricted by travel visa requirements. Detailed results of the WGS and ISC-SLG analyses are provided in S1 Database. Of the 204 isolates, 7 originated from patients who were treated a second time, and that showed the same genotype as found in the first disease episode. When these 7 are not taken into account, as they do not represent independent data points, we found 33 ISC001, 16 ISC003, 33 ISC004, 15 ISC005, 43 ISC006, and 8 ISC009 isolates, while 49 could not be classified (6/93 analyzed with WGS, 43/104 analyzed with ISC-SLG).

**Fig 2:**
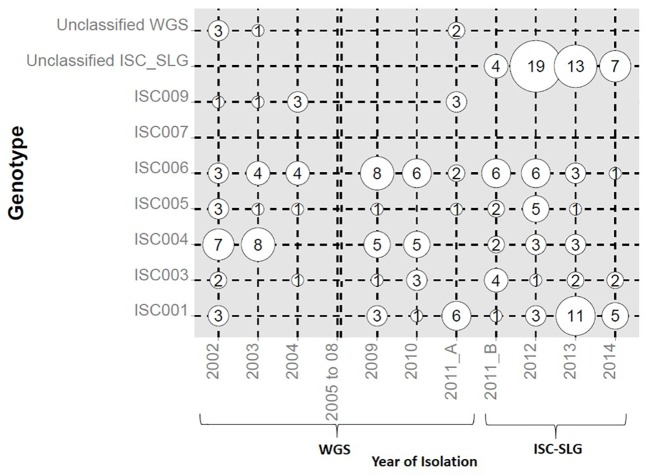
Year-wise distribution of genotypes. Years of parasite isolation are at the bottom, genotypes were identified by WGS or ISC-SLG, as indicated. No samples were available from 2005 to 2008, indicated by a double vertical line. Genotypes are shown on the left. The diameter of bubble plots represents the number found in each year. When isolates were derived from different disease episodes of the same patient, they are shown only if the genotypes in these episodes were different, thereby excluding 5/98 and 2/106 isolates characterized by WGS and ISC-SLG respectively.

Fig 2 illustrates the distribution of genotypes over the period of sampling. Genotypes ISC001 and ISC003-ISC006 were present in most sampled years, with some exceptions. But even when they were not found in a particular year, they reappeared later on, proving that all of them circulated continuously in the region. For a given year, the most abundant classified genotype varied: ISC004 in 2002–2003, ISC006 from 2004 to 2012, and ISC001 in 2013–2014. ISC009 was the rarest genotype and was not identified after 2011 because no ISC-SLG test is available. ISC007 was not found in any sample.

**Fig 3:**
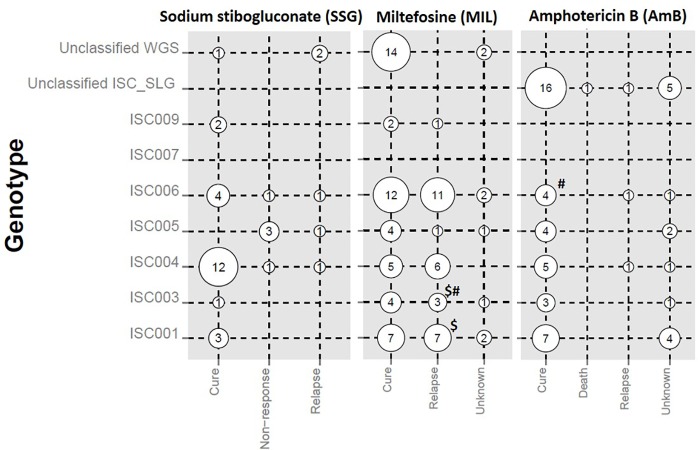
Genotype distribution in relation to treatment outcome for all episodes for which treatment was completed. Outcomes of three drugs (SSG, MIL, AmB) are given at the bottom, genotypes are shown on the left. Cure: treatment completed, absence of clinical sign and symptoms of VL until six months for SSG and AmB, and one year for MIL; Death: death of patient during course of treatment; Non-response: persisting clinical signs and symptoms of VL, and positive bone marrow smear after treatment; Relapse: reappearance of disease symptoms and/or positive bone marrow smear after initial cure and during a 12 month follow-up. $# denotes two MIL treated patients who relapsed but presented infection with a different genotype before and after relapse: $, BPK519; # BPK676, see S1 Database for details. The diameter of bubble plots is proportional to the number of the genotypes for each treatment outcome.

The distribution of genotypes according to the treatment outcome of patients with three different drugs (SSG, MIL and AmB) is shown in Fig 3, whereby only episodes where treatment was completed are included. With respect to SSG, treatment failure was observed only in ISC004-ISC006, but most patients infected with ISC004 and ISC006 were cured. In contrast, none of the patients infected with ISC005 cured after SSG treatment. For MIL, relapse was observed for the 6 identified genotypes but never for the unclassified ones. Half of four MIL treated relapsing patients for which genotyping was done at relapse stage, showed the same genotype before and after relapse. Two of them however did not: BPK519 and BPK676 were initially infected with ISC003, but at the time of relapse respectively ISC001 and ISC006 were isolated. The three cases of AmB relapse were associated with ISC004, ISC006, and an unclassified genotype.

**Fig 4:**
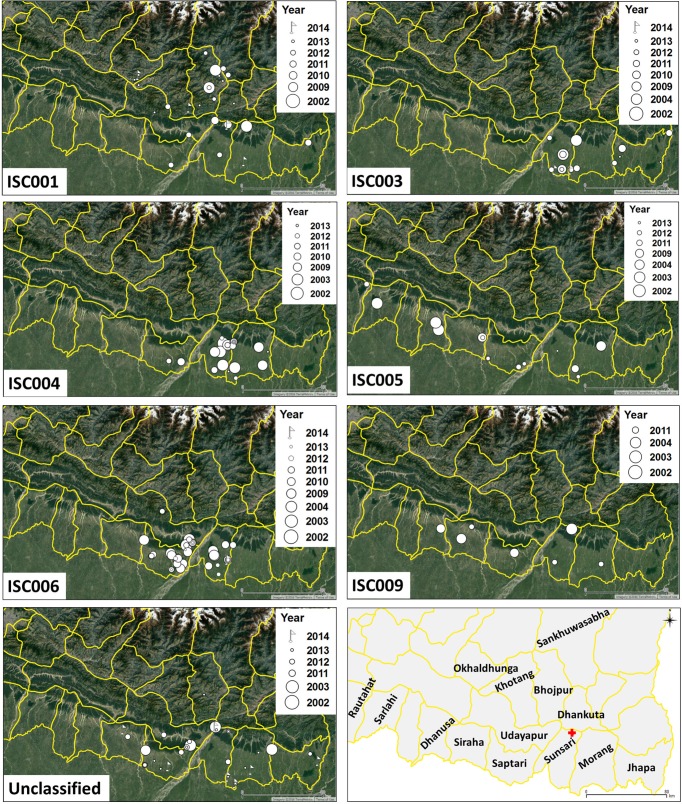
Spatial-temporal distribution of each *L*. *donovani* genotype. The sampling districts (South-East Nepal) are shown at the bottom right panel. BPKIHS (Dharan) is indicated with a red cross. One ISC001 isolate of 2014 originated from the Banke district, which is not shown because it is located in western Nepal. Data can be viewed interactively from https://microreact.org/project/SNP_Genotypes_Nepal

VL patients included in this study were from 15 administrative districts. Fig 4 shows the presumed geographical origin of typed parasites over time, with the assumption that patients got infected in their home village. An overview of the numbers per year and per district is given in S1 Table. Seven patients for which identical genotypes were recovered before and after relapse were included only once in this analysis. Genotype ISC001 was mostly present in hill districts such as Bhojpur, Dhankuta, Khotang, Okhaldhunga, Sankhuwasabha, and Udayapur. Other genotypes were unevenly distributed in the lowland area (Terai). The highest number of different genotypes was found in the three districts Morang, Sunsari and Saptari.

## Discussion

Several *Leishmania* populations have been documented to circulate in the Indian Subcontinent [10]. Keeping track of their distribution over time is of particular importance for parasite elimination by control and treatment measures. Indeed, tracking the spread of populations allows tracing parasite movements from one area to another, assisting in monitoring control efforts. In addition, parasites from different populations could react differently to treatment, be carried by different vectors, or differ in response to vaccines should they become available. Identifying *L*. *donovani* populations in the ISC is however challenged by the fact that they are genetically highly homogeneous: WGS revealed no more than 2418 SNPs in 191 isolates of the core population [10]. Previously used methods such as microsatellites, which are effective in other areas in the world, have shown limited value in this region [5]. Data from WGS offered the additional resolution needed for distinguishing different populations based on single nucleotide polymorphisms [10]. Although WGS could be used for large-scale epidemiological surveys or clinical studies, it remains currently too expensive, has limited availability, and requires too much bio-informatics analysis time. The ISC-SLG method presented in this paper partly solves these limitations, as it requires only PCR and classic Sanger sequencing methods, which are far easier to perform and interpret than WGS, at a significantly smaller cost.

As a proof-of-principle, we investigated the geographic spread of different parasite genotypes over a period of more than 10 years (2002–2014), combining data from WGS and ISC-SLG of *L*. *donovani* in Nepal. Based on these data we could show that particularly ISC001 has a different geographic distribution as compared to the other genotypes, and is present predominantly in hilly areas. This could be related to a local epidemic, which is corroborated by a recent outbreak investigation showing that transmission does take place at higher altitudes [20]. ISC001 is genetically very different from parasites belonging to the main population endemic in the Terai lowland, and shows different phenotypic features, hence its epidemiological follow-up is highly relevant. No obvious pattern could be seen for the other genotypes, which are found in the Terai (lowland). As this analysis was merely a proof-of-concept, no firm conclusions should be drawn. The data collection was not set up for such precise monitoring, and only confirmed VL cases visiting the BPKIHS medical services were included. In addition, the sampling should be extended to other countries of the ISC.

An assessment was presented on differential treatment response from the different genotypes. Apart from ISC005, which systematically did not react to SSG, no obvious patterns were seen. The treatment failure of ISC005 infected patients does not come as a surprise, because all parasites of this population have a defective aquaglyceroporin transporter [21]. This transporter is needed for uptake of trivalent antimonials, which are the active derivatives of pentavalent antimonial drugs such as SSG. It is exactly the underlying 2-nucleotide insertion responsible for the inactivating frameshift that was used for identification of ISC005. As for the other treatments, AmB was effective in all genotypes, and MIL frequently resulted in relapse, also across genotypes. Regarding the latter, no relapse was seen in unclassified genotypes, which remains to be elucidated: identification of these samples is needed, as well as a sufficiently powered study.

In two patients, different genotypes were detected at the onset of disease and at relapse stage after MIL treatment. Several scenarios could explain this. First, the patients might have been simultaneously infected with 2 different genotypes initially, of which one outgrew the other during culturing. After treatment, the first detected parasite could have been eliminated, allowing the other to be isolated at the time of relapse. Alternatively, the patients might have been re-infected with a different genotype, causing the relapse.

As WGS-based methods can be considered the gold standard for identifying parasite populations because they use nearly all nuclear sequence information, our study can assess the power of alternative methods based on single or few genes. A previous report, including 31 strains from our analysis, classified Nepalese genotypes with kinetoplast DNA mini-circles, microsatellites, cysteine proteinase B, glycoprotein 63, and the hydrophilic surface protein B [9]. This analysis identified 14 kDNA and 8 nuclear genotypes, which in general correlated well with WGS-defined genotypes (data provided in S1 Database), even though there was no perfect one-to-one relationship. This could be explained by the fact that microsatellites tend to suffer from homoplasy [22], mini-circles are highly variable and difficult to analyze in a reproducible manner, and genetic homogeneity calls for analysis of many loci in parallel.

We applied ISC-SLG on parasite cultures, but so far not on clinical samples. Nevertheless, the analytical sensitivity of the assays was between 0.2 and 2 pg, or roughly 1 to 10 parasite genomes, which makes the assay at least theoretically usable on VL samples with sufficient parasite load, such as bone marrow aspirates, spleen/liver biopsies, and even blood [23]. In addition, also tissue from Post-Kala-azar dermal leishmaniasis (PKDL) patients was shown to surpass this threshold [24]. Also, currently efforts are ongoing to specifically select or amplify low abundant parasite DNA from clinical samples, which may provide new opportunities to extract useful genetic information from these [25].

Improvement of ISC-SLG could involve the use of multiplex and real-time PCR. Currently, each genotype requires a different PCR, and these could be combined in a single tube by multiplexing to increase speed and reduce cost. Moreover, as illustrated in this paper, analysis can be done in a sequential manner, whereby samples are first tested for the most abundant genotype(s) instead of running all assays simultaneously. Further, to enhance throughput and compatibility with existing infrastructure in low-resource settings, sequencing could be avoided by using fluorescent real-time PCR probes as shown by Feehery et al. and Tyagi et al. [26,27].

It should be noted that ISC-SLG can detect documented genotypes/populations, but does not allow identification of new ones or alternative genotypes showing the same polymorphism as used in the assay. Indeed, 42% of tested parasite isolates could not be classified. These could either represent hitherto undetected genotypes, or populations for which no ISC-SLG test was developed or used. Nevertheless, highly useful information can be obtained even by tracking half of the circulating genotypes, as it can provide insight into the way of spreading, and hint to invasion of new genotypes in a particular area. Hence, WGS remains complementary to our method, allowing to investigate new genotypes and serving as a basis for developing additional ISC-SLG assays.

In conclusion, ISC-SLG was successfully applied to cultured isolated parasites from various locations in Nepal. The method needs further improvement, but is already in its current version a practical tool for use in clinical studies and epidemiological surveys. As such it contributes to “research on epidemiology and transmission dynamics of VL”, one of the key research priorities for visceral leishmaniasis elimination recently suggested by Singh et al. [28]. Applying our technique in country-wide systematic epidemiological surveys would help to better understand and control leishmaniasis in Nepal. The method could easily be extended to additional genotypes circulating in other regions of the ISC, in support of the Kala-azar elimination program.

## Supporting information

S1 Database Detailed information of all studied strains. The MS Excel file contains detailed information on geographic origin, treatment regimens, and genotyping from all 204 disease episodes analyzed in this study.
(XLS)

S1 Table Year and district-wise distribution of *L*. *donovani* genotypes. For each year and per district, the number of isolates is given. The 7 disease episodes from patients from whom the same genotype was recovered in the first and second episode were not counted, as these probably do not represent new infections.
(PDF)
