# Supplementary material for: Single locus genotyping to track Leishmania donovani in the Indian subcontinent: Application in Nepal
Source: PLoS Negl Trop Dis. 2017 Mar 1;11(3):e0005420. doi: 10.1371/journal.pntd.0005420 (PMC5348045; doi:10.1371/journal.pntd.0005420)
Supplement: S1 Table — For each year and per district, the number of isolates is given. The 7 disease episodes from patients from whom the same genotype was recovered in the first and second episode were not counted, as these probably do not represent new infections. (PDF) [file pntd.0005420.s002.pdf]

Table S1: Year and District-wise distribution of *L. donovani* genotypes

Year of parasite isolate	District	Genotype							Total	Yearly total
		ISC001	ISC003	ISC004	ISC005	ISC006	ISC009	Unclassified		
2002	Bhojpur	2	0	0	0	0	0	0	2	22
	Dhanusa	0	0	0	2	0	0	0	2	
	Jhapa	0	0	0	0	0	0	1	1	
	Morang	1	1	1	0	0	0	0	3	
	Saptari	0	0	0	0	2	0	1	3	
	Sarlahi	0	0	0	1	0	0	0	1	
	Sunsari	0	1	6	0	1	1	1	10	
2003	Morang	0	0	2	1	0	0	0	3	15
	Saptari	0	0	0	0	1	0	1	2	
	Siraha	0	0	0	0	1	1	0	2	
	Sunsari	0	0	6	0	2	0	0	8	
2004	Dhanusa	0	0	0	0	0	1	0	1	9
	Saptari	0	0	0	0	4	2	0	6	
	Siraha	0	0	0	1	0	0	0	1	
	Sunsari	0	1	0	0	0	0	0	1	
2009	Jhapa	0	1	0	0	0	0	0	1	18
	Morang	0	0	4	1	0	0	0	5	
	Saptari	0	0	1	0	5	0	0	6	
	Sunsari	3	0	0	0	3	0	0	6	
2010	Jhapa	1	1	0	0	0	0	0	2	15
	Morang	0	1	3	0	1	0	0	5	
	Saptari	0	0	0	0	5	0	0	5	
	Sunsari	0	1	2	0	0	0	0	3	
2011	Jhapa	0	1	0	0	0	0	0	1	33
	Khotang	1	0	0	0	1	0	0	2	
	Morang	0	1	0	0	1	1	1	4	
	Rautahat	0	0	0	1	0	0	0	1	
	Sankhuwasabha	3	0	0	0	0	0	0	3	
	Saptari	2	0	1	1	5	0	2	11	
	Siraha	0	0	0	0	0	1	1	2	
	Sunsari	1	2	1	1	1	1	1	8	
	Udayapur	0	0	0	0	0	0	1	1	
2012	Bhojpur	3	0	0	0	0	0	0	3	37
	Jhapa	0	0	0	0	0	0	1	1	
	Morang	0	0	2	1	0	0	15	18	
	Saptari	0	0	0	3	2	0	1	6	
	Siraha	0	0	0	1	0	0	0	1	
	Sunsari	0	1	1	0	4	0	2	8	
2013	Bhojpur	3	0	0	0	0	0	1	4	33
	Dhankuta	1	0	0	0	0	0	0	1	
	Jhapa	0	2	0	0	0	0	0	2	
	Khotang	1	0	0	0	0	0	0	1	
	Morang	0	0	3	0	0	0	6	9	
	Okhaldhunga	5	0	0	0	0	0	0	5	
	Rautahat	0	0	0	0	0	0	1	1	
	Saptari	0	0	0	0	3	0	3	6	
	Sunsari	0	0	0	1	0	0	2	3	
	Udayapur	1	0	0	0	0	0	0	1	
2014	Banke	1	0	0	0	0	0	0	1	15
	Jhapa	0	0	0	0	0	0	1	1	
	Khotang	1	0	0	0	0	0	0	1	
	Morang	1	1	0	0	0	0	3	5	
	Okhaldhunga	1	0	0	0	0	0	0	1	
	Saptari	0	0	0	0	0	0	1	1	
	Siraha	0	0	0	0	0	0	1	1	
	Sunsari	1	1	0	0	1	0	1	4	
	Total	33	16	33	15	43	8	49	197	
